# The Effects of Lemon Balm (*Melissa officinalis* L.) Essential Oil on the Stress Response, Anti-Oxidative Ability, and Kidney Metabolism of Sea Bass during Live Transport

**DOI:** 10.3390/ani12030339

**Published:** 2022-01-29

**Authors:** Qi Wang, Jun Mei, Jing Xie

**Affiliations:** 1 College of Food Science and Technology, Shanghai Ocean University, Shanghai 201306, China; wangqi4459@163.com; 2 National Experimental Teaching Demonstration Center for Food Science and Engineering, Shanghai Ocean University, Shanghai 201306, China; 3 Shanghai Aquatic Products Processing and Storage Engineering Technology Research Center, Shanghai Ocean University, Shanghai 201306, China; 4 Shanghai Engineering Research Center of Aquatic Product Processing & Preservation, Shanghai Ocean University, Shanghai 201306, China; 5 Collaborative Innovation Center of Seafood Deep Processing, Ministry of Education, Dalian 116034, China

**Keywords:** anesthetic, live transport, lemon balm, oxidative stress, sea bass

## Abstract

**Simple Summary:**

The greatest challenge of long-distance live transport of fish is high mortality caused by stress and oxidative damages. In this study, the effects of lemon balm (*Melissa officinalis* L., MO) on the stress response of sea bass were evaluated. Sea bass were treated with different concentrations of MO (10, 20, 40 mg/L, respectively) and were transported for 72 h in transport boxes. The results of this study indicated that the level of cortisol, glucose, lactic acid, heat shock proteins, catalase, myeloperoxidase, glutathione peroxidase, uric acid, and urea nitrogen of samples treated with MO were lower than the control. The sample treated with 40 mg/L MO showed higher antioxidant capacity. In conclusion, the effect of MO on alleviating stress responses was similar to MS-222 and eugenol.

**Abstract:**

This study was conducted to enhance the viability and alleviate the oxidative stress response using MO for sea bass during live transport. Six experimental groups were designed, and the effects of the physiological responses of MO were evaluated in comparison with MS-222 and eugenol. The physiological stress levels, proprotein convertase subtilisin/kexin type 9 (PCSK-9), antioxidant enzyme activities, and kidney parameters of blood serum were determined. It was found that cortisol level, glucose (Glu), lactic acid (LD), heat shock proteins (HSPs), catalase (CAT), myeloperoxidase (MPO), glutathione peroxidase (GSH-Px), uric acid (UA), and urea nitrogen (BUN) in the MO-treated samples were lower than that of the control (133.72 ng/L); however, the total antioxidant capacity (T-AOC) was higher after 72 h of the simulated live transport. The ability to resist oxidative stress increased along with the increase in the MO concentration in the water during live transport, which was similar to the results of MS-222 and eugenol treatment. In conclusion, MO, acting as a kind of novel sedative and anesthetic, can be used to improve the oxidative system and survival rate during live transport. The results of this study provide a reference for enhancing animal welfare and anti-oxidative stress ability, reducing mortality and the stress response during live fish transport.

## 1. Introduction

Live fish transport is becoming increasingly important in aquatic product trade and fish are put in bags or containers and then transported alive [1]. However, fish are vulnerable to aberrant environmental stress factors and display robust neuroendocrine and physiologic parameter changes and a stress response [2,3]. Many studies have been conducted on environmental stress of live fish, such as tissue hypoxia stress [4,5], water quality stress [6,7,8], salt concentration and temperature stress [9,10], physical handling stress, etc. [11]. Therefore, the toughest challenge during live fish transportation is minimizing the stress response and sensitivity induced by environmental changes to obtain a high survival rate. The stress response is a complex regulatory mechanism that occurs during live fish transport, which can be divided into three levels [12]. The first stress response is a neuroendocrine reaction, in which the hormones of fish are released along with an increase in cortisol, adrenaline, and other hormones. Nestor et al. [13] found that capture, transport, and high stocking density caused high cortisol levels during live fish transport. The secondary stress response consists of changes in energy metabolism, blood physiology, and biochemistry and immune regulation caused by neuroendocrine reactions. The tertiary stress response affects performance [14]. Wang et al. [15] showed that the function of immune and antioxidative parameters of juvenile tiger puffer (*Takifugu rubripes*) were affected after short-term live transport and recovered at 168 h.

At present, the common methods used for live transport include optimization of transportation technology, upgrade of facilities, and addition of anesthetics or sedatives to the transport water to improve the efficiency of live transport [16,17,18]. Some anesthetics, such as tricaine methanesulfonate (MS-222) and eugenol, have been developed. Essential oils have been used in live fish transport and have a similar efficiency to chemical anesthetics [19,20]. The addition of anesthetics and essential oils to transport water can temporarily block sensory (afferent) nerve conduction to relieve pain, discomfort, struggle, and physical energy consumption induced by stress [21]. Lemon balm (*Melissa officinalis* L., MO) is also known as American mint and wild bergamot. The main ingredients of lemon balm are aldehydes, terpenes, and phenolic compounds, such as citral, rosmarinic acid, and flavonoids [22,23]. Its mechanism of action involves rosmarinic acid (RA), a compound that inhibits γ-aminobutyric acid (GABA) transaminase activity and slows the degradation of GABA. RA is able to cross the blood–brain barrier and acts on GABA in the brain, thereby maintaining the stability of GABA concentrations in living organisms [24]. At present, some scholars have applied plant essential oil to transport water in live fish transport. Boaventura et al. [25] confirmed that lophiosilurus alexandri (*Siluriformes*, *Pimelodidae*) can be stabilized by treatment with 90 and 150 mg/L essential oil of *Ocimum gratissimum* L. (EOOG) in transport water, resulting in a decrease in the metabolic rate of fish and an increase in the antioxidant stress ability. Khumpirapang et al. [20] and Rodrigues et al. [19] developed *Alpinia galanga* oil (AGO) and canela-amarela (*Nectandra grandiflora*) essential oil as novel anesthetics for use during live fish transport. Essential oil is a promising natural source of alternative sedatives and anesthetics for fish. In the present study, sea bass (*Lateolabrax japonicus*), an edible marine fish, were used as experiment subjects. This study evaluated the negative effects of live fish transport on sea bass by investigating the influence of the addition of MO and two kinds of anesthetics to the transport water. Sea bass were transported in cold water (12 °C) and treated with different concentrations of MO, MS-222, and eugenol. The objective of this study was to exploit a novel and effective sedative and anesthetic to enhance animal welfare and the survival rate of live edible marine fish and reduce the stress effects and oxidative damage.

## 2. Materials and Methods

### 2.1. Preparation of Sea Bass

This experiment followed the principles and guidelines established by the animal care and use committee of Shanghai Ocean University (SHOU-DW-2021-67). Sea bass (500 ± 120 g, 39 ± 1 cm) were purchased from the local market in Luchao Port town (Shanghai, China) and transported to the laboratory in live fish transport boxes within 30 min. The temperature in the facility from which the fish were purchased was 20~22 °C. All of the fish were healthy and unhurt, and fish were temporarily cultured for 36 h without feeding [26]. The temporarily cultured water had the following parameters: the density of temporary culture was 20 g/L, the temperature of the water was 20~22 °C, the salinity was 16, the dissolved oxygen was 4~6 mg/L, and the pH was 7.5~8.5. After temporary culture, the temperature was dropped from 20~22 °C to 12 °C at a rate of 3 °C/h. The temperatures used in this study are within the natural tolerance range of this species [3,18,27]. When the temperature of the transport water reached 12 °C, the live transport experiment started.

### 2.2. Live Transport Experimental Design

The optimum concentration was obtained from a pre-experiment, and the MO and eugenol solution were prepared in a mixture of alcohol and tween-80 (200:1, *v*/*v*). Six groups were used in this study. The concentrations of MO in the transport water were 10 (10 MO), 20 (20 MO), and 40 (40 MO) mg/L; MS-222 was 30 mg/L (MS-222) [28]; and eugenol was 20 mg/L (Eugenol) [29], respectively. The character of MS-222 was weakly acidic, and the pH value range of the transport water after adding 30 mg/L MS-222 was 7.0~8.0. The sea bass samples in transport water to no agents were added were considered as the control. Sea bass were stocked and transported in live transport boxes with the same volume of cold water (12 °C) to which different concentrations of MO and two kinds of anesthetics were added to in advance [30]. The volume of the boxes was 108 L. The sea bass underwent simulated transport for 72 h and the density of live transport was 250 g/L. The water parameters were the same as the temporarily cultured water. The number of fish in each box per group was 30, and a total of 180 fish were used in the experiment. MO was purchased from Gaodao essential oil trading company (Chongqing, China). The main components were citral (44.9%), geraniol (21.1%), citionella (15.4%), citronellol (6.3%), rosmarinic acid (4.1%), and D-limonene (2.3%). The simulated transport was as follows: 1 h on a B-level road (80 km/h) →4 h on an A-level road (100 km/h) →1 h on a B-level road (80 km/h), repeated 12 times. Three sea bass were randomly selected for sampling after 2, 4, 6, 8, 10, and 12 cycles. After simulated transport for 72 h, the sea bass samples were recovered in cultured water at room temperature. Eventually, the survival rates were determined [16,31]:

$$\mathrm{Survival}\;\mathrm{rate}=\frac{\mathrm{Survival}\;\mathrm{number}}{\mathrm{Sample}\;\mathrm{number}}\times 100\%$$

### 2.3. Pretreatment of Samples

The sea bass were stunned with ice water for 15 min and then killed. Ice did not touch the fish during this period. The blood of sea bass was taken from the tail vein without anticoagulant. The blood was stored at 4 °C for 2 h, and then centrifuged at 10,614× *g*, 4 °C for 5 min, and the liquid supernatant (serum) was collected. The serum was stored at −80 °C before use for index determination.

### 2.4. Determination of the Physiological Stress Level

Glucose and lactic acid in serum were measured using commercial kits (Jiancheng Bioengineering Institute, Nanjing, China). The methods used to detect cortisol and heat shock proteins was enzyme-linked immunosorbent assay (ELISA), and determination of heat stress protein and cortisol antibody-coated pore plates, respectively. The absorbance (OD value) was measured at 450 nm with a microplate reader, and the sample concentration was calculated. Commercial fish ELISA kits for cortisol and heat shock proteins were supplied by Jiancheng Bioengineering Institute (Nanjing, China);

### 2.5. Determination of Proprotein Ponvertase Subtilisin Kexin Type 9 (PCSK-9)

The method used to detect PCSK-9 was enzyme-linked immunosorbent assay (ELISA) and determination of alanine aminotransferase (PCSK9) coated on a pore plate. The absorbance (OD value) was measured at 450 nm with a microplate reader, and the sample concentration was calculated. Fish ELISA kits for PCSK-9 were supplied by Jiancheng Bioengineering Institute (Nanjing, China).

### 2.6. Determination of Antioxidant Enzyme Activities

Catalase activity (CAT) in the serum was calculated by measuring change in the yellow complex produced by the interaction of hydrogen peroxide and ammonium molybdate. The vitality unit is expressed in U/mL. The activity of myeloperoxidase (MPO) was calculated by the yield of the yellow compound produced by hydrogen donor anisidine, and the vitality unit is expressed in U/mL. CAT and MPO were quantified by commercial kit assays (Jiancheng Bioengineering Institute, Nanjing, China). The consumption of reduced glutathione (GSH) in an enzymatic reaction was measured and the activity of glutathione peroxidase (GSH-Px) was calculated by the speed of the enzymatic reaction. Glutathione peroxidase (GSH-Px) activity was determined by referring to Boaventura et al. [25]. Total antioxidant capacity (T-AOC) in serum was measured using commercial kits (Jiancheng Bioengineering Institute, Nanjing, China).

### 2.7. Determination of Kidney Function Index

Uric acid (UA) and blood urea nitrogen (BUN) in serum were determined by Jia et al. [32].

### 2.8. Statistical Analysis

The one-way ANOVA-Duncan test program in SPSS 21.0 software was used for multiple comparisons. The Levene test was used to check the homogeneity of the samples before applying Duncan. The results are expressed as means ± SD, and the significance threshold was 0.01. Origin software was used to make graphs.

## 3. Results and Discussion

### 3.1. Survival Rates of Sea Bass

The survival rates during and after long-distance live transport of sea bass were recorded and are presented in Table 1. The survival rates of the fish treated with MO or anesthetics were higher than that of the control (50%) after 12 h of recovery. The survival rate of samples in the 20 MO and 40 MO groups was 96% after transport and recovery. The survival rate of samples in the MS-222 group was 100% and the survival rate of samples in the eugenol group was 95%. No fish from the 20 MO, 40 MO, and MS-222 treatment groups died during the period of recovery.

### 3.2. Physiological Stress Level

**Figure 1:**
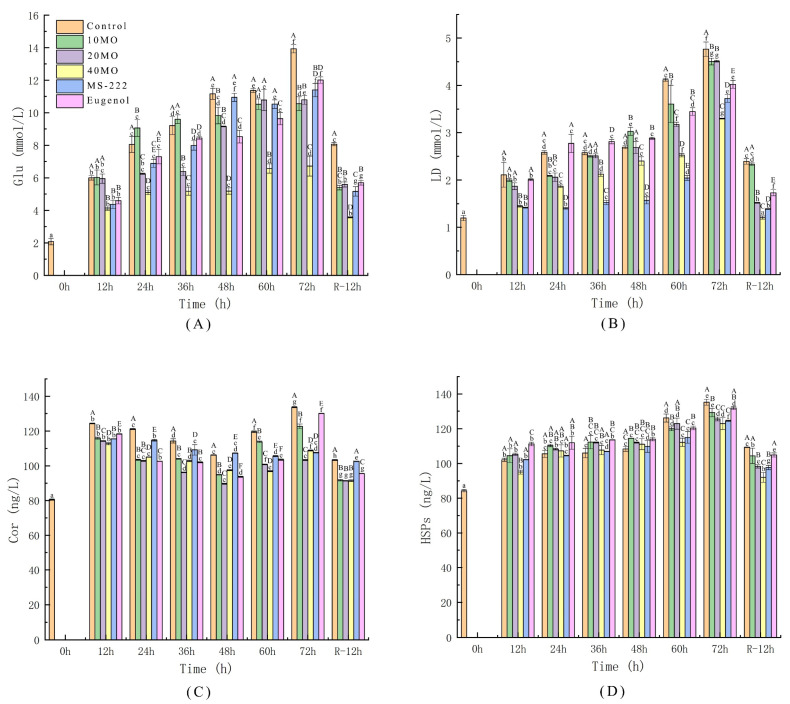
The changes in Glu (**A**), LD (**B**), Cor (**C**), and HSPs (**D**). 10 MO: 10 mg/L MOEO, 20 MO: 20 mg/L MOEO, 40 MO: 40 mg/L MOEO, MS-222: 30 mg/L MS-222, eugenol: 20 mg/L eugenol, control: no agent addition. Vertical bars indicate the standard deviation; Means with different lowercase letters indicate a significant difference between time intervals within each group (*p* ≤ 0.01) while different capital letters indicate significant differences between groups in each time interval (*p* ≤ 0.01).

As shown in Figure 1C, cortisol in sea bass samples increased at the beginning and then decreased during live transportation. Cortisol levels significantly increased from 0 to 12 h. The level of cortisol reflects the stress response of fish [33]. The increase in the cortisol level suggests that fish’s neuroendocrine system exerted a primary response and secreted a large amount of cortisol during the initial stage of environmental stress. Then, the level of cortisol decreased with transport time, which indicated that fish gradually adapted to the initial stress during live transport. However, the accumulation of excreted waste increased the stress response of sea bass. The fish presented a secondary stress response after transport for 60 h and the stress indices reached a peak value after transport for 72 h, which is consistent with the research of Wang et al. and Vanderzwalmen et al. [34,35], who found that MO-treated samples demonstrated lower cortisol levels than other samples during live transport. The cortisol level decreased dramatically after 12 h of recovery, and the cortisol level of the MO treatment groups was the lowest in comparison with those of the other groups with no significant difference.

Muscle and liver store energy in the form of glycogen, and blood in the form of glucose, which are important energy substances required for the various life activities of fish. Blood glucose levels can affect fish’s stress response by modulating cortisol release and glucose homeostasis to affect glycogen metabolism and gluconeogenesis [36]. Lactic acid is produced by glycolysis in the presence of an insufficient oxygen supply. The stress response evoked during live fish transportation caused anaerobic muscle activity and the formation of lactic acid both in the muscle and blood [37]. It can be observed from Figure 1A, B that the glucose and lactic acid levels of each treatment groups increased along with the increase of the transport time and reached a peak value after 72 h of live transport and then decreased sharply after 12 h of recovery. The lactic acid level of 40 MO returned to the 0 h level with no significant difference after 12 h of recovery. This trend could be ascribed to the increase in the glycogen level to combat the stress response and the body performing anaerobic respiration to produce large amounts of lactic acid induced by the low temperature. At the same time, the increase in the serum cortisol level promoted the generation of glycogen, resulting in an increase in serum glucose. These results are close to the values presented by Liu et al. and Zhao et al. [38,39]. It was observed that the glucose and lactic acid levels of the control were the highest and those of the sea bass in the group treated with 40 MO were the lowest during the whole simulated transportation. The glucose level in the serum gradually decreased along with the increase in the MO concentration and the glucose level of sea bass in 40 MO was lower than those of other groups. Similar trends were encountered for the change in the lactic acid level for all treatment groups, with differences between the values. It was indicated that the energy consumption of sedated and anesthetized fish was lower in comparison with that of the control, and the levels of glucose and lactic acid in the samples of the 40 MO treatment group were the lowest in comparison with those of other groups.

The heat shock protein level is low and stable for fish under normal conditions; however, the heat shock protein level increases to different degrees to protect the body tissues and organs from damage when fish are exposed to stress [40]. Figure 1D shows an increase trend in heat shock proteins among each treatment groups during the whole live transport process. After transport for 72 h, heat shock proteins in the control displayed the highest level (135.20 ng/L) with no significant difference to that of the eugenol group (131.86 ng/L) while the 40 MO groups showed the lowest level (122.91 ng/L) with no significant difference with that of the 20 MO group (125.53 ng/L) and MS-222 group (124.41 ng/L). As compared with the control, the fish stress response of the groups treated with MO and anesthetics was relatively light. With the increase in the MO concentration in the transport water, the production of heat shock proteins was relatively low during live sea bass transport.

The sedated and anesthetized fish showed a lower physiological stress response than that of the control, especially fish in the 40 MO treatment group, which showed a minimum change in the serum stress indexes. The application of MO to the transport water mitigated the stress response and improved the antistress ability of sea bass.

### 3.3. PCSK-9

**Figure 2:**
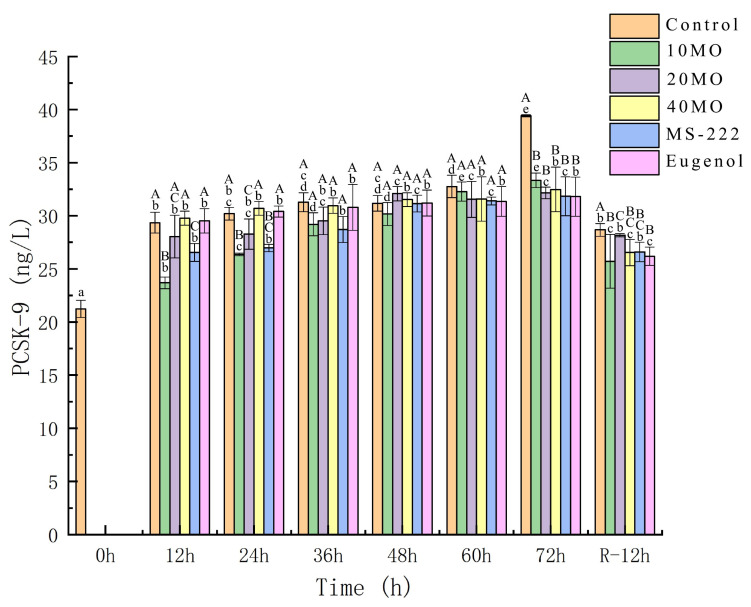
The activity changes in PCSK-9. 10 MO: 10 mg/L MOEO, 20 MO: 20 mg/L MOEO, 40 MO: 40 mg/L MOEO, MS-222: 30 mg/L MS-222, eugenol: 20 mg/L eugenol, control: no agent addition. Vertical bars indicate the standard deviation; Means with different lowercase letters indicate a significant difference between the time intervals within each group (*p* ≤ 0.01) while different capital letters indicate significant differences between the groups in each time interval (*p* ≤ 0.01).

PCSK-9 is also known as neural apoptosis-regulated convertase (NARC1) and is a newly identified subtilase belonging to the peptidase S8 subfamily. Additionally, PCSK9 is suggested to participate in immunoregulation by modulating cytokine production and indirectly affecting the apoptosis rate and blood cholesterol levels. Activated PCSK-9 plays a role in the promotion of apoptosis [41,42,43]. It can be observed from Figure 2 that the PCSK-9 activity in each group increased dramatically after 12 h of transport, which indicated that fish suffered stress responses and cell apoptosis during the initial simulated transport and PCSK-9 activity was activated in the organisms. There was a significant increase in PCSK-9 activity in the control (39.42 ng/L) after 72 h of transport due to the persistent stress response. The activity of PCSK-9 decreased dramatically after 12 h of recovery. The results suggested that a decrease in the PCSK-9 activity indicated alleviation of the stress response for sea bass after 12 h of recovery; however, the stress response was not completely eliminated. It is proved that both anesthetics and MO can be used to protect the body from injury, and the results of this study demonstrate the efficacy of anesthetics and sedatives with MO.

### 3.4. Antioxidant Enzyme Activities

The activity of antioxidant enzymes in fish was activated in order to resist lipid peroxidation in the stress environment. SOD converts O_2_^−^ to O_2_ and H_2_O_2_ first, and then CAT catalyzes H_2_O_2_ to harmless H_2_O and O_2_ at random. As an important peroxidase decomposition enzyme, GSH-Px catalyzes other harmful peroxides in the body and reduces toxic peroxides to non-toxic hydroxyl compounds, to protect the structure and function of cell membranes of living organisms [44,45]. MPO is a marker of neutrophil function and activation, and the peroxidase activity of MPO generates hypochlorite to kill microbes and inactivate inhibitors of lytic enzymes. Next, neutrophil leukocytes are released to degrade material in their vicinity [46]. Therefore, these enzymes make up the body’s antioxidant defense system.

**Figure 3:**
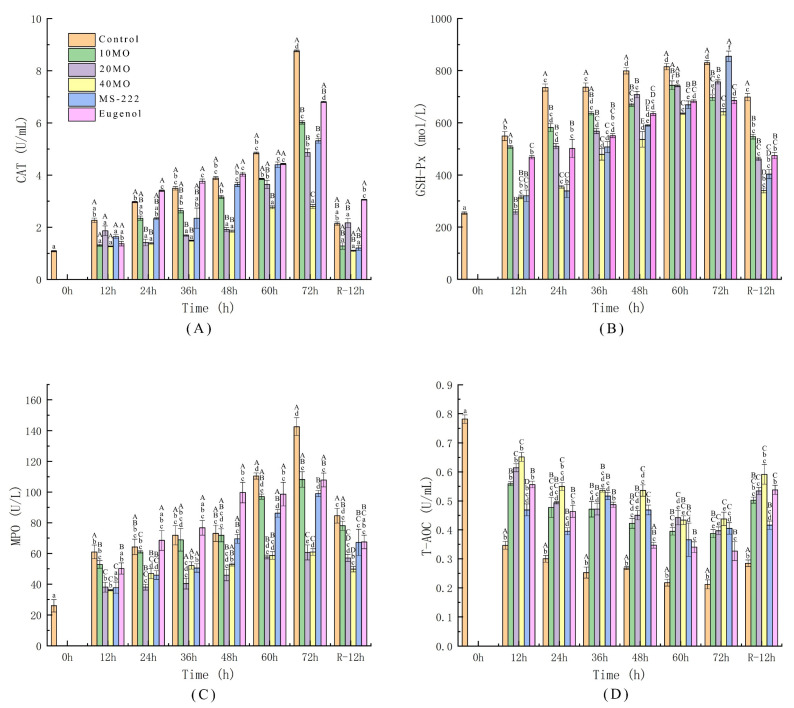
The activity changes in CAT (**A**), GSH-Px (**B**), MPO (**C**), and T-AOC (**D**). 10 MO: 10 mg/L MOEO, 20 MO: 20 mg/L MOEO, 40 MO: 40 mg/L MOEO, MS-222: 30 mg/L MS-222, eugenol: 20 mg/L eugenol, control: no agent addition. Vertical bars indicate the standard deviation; Means with different lowercase letters indicate a significant difference between the time intervals within each group (*p* ≤ 0.01) while different capital letters indicate significant differences between groups in each time interval (*p* ≤ 0.01).

It can be observed from Figure 3A–C that the CAT, GSH-Px, and MPO activity in the samples of each group showed a slow growth trend during live transport and presented a sharp increase in CAT and MPO activities in the control (*p* ≤ 0.01) after 72 h of transport, which indicated that oxidative stress displayed an exponentially increasing trend along with the increase in the transport time. However, Karu et al. [47] and Zeng at al. [48] found that the CAT activity in fish blood did not change significantly and the activity of CAT increased in fish liver. These changes may indicate a specific ability of fish to protect the tissue and organs of the body under low-temperature conditions. After transport for 72 h, the CAT, GSH-Px, and MPO activities of samples in the 40 MO group showed the lowest level in comparison with the other groups, and the antioxidant efficiency was positively correlated with the concentration of MO. The GSH-Px activity in the samples treated with MS-222 demonstrated a sharply increasing tendency to the highest level (855.14 mol/L) after 72 h of transport. The enzymatic activity in the samples treated with 40 MO (642.16 mol/L) presented lower activity than the samples treated with anesthetics (MS-222: 855.14 mol/L, eugenol: 685.41 mol/L) and the control (830.27 mol/L). This may be because anesthetized fish were woken up for anesthetic consumption and presented a stress response, or different individuals showed various oxidative stress responses. After 12 h of recovery, the antioxidant enzyme activities in each group demonstrated an obvious downward trend. The enzymatic activity of the samples in the 40 MO group was the lowest (340.54 mol/L) compared with the samples in the other groups, which meant that the addition of 40 mg/L MO to the transport water alleviated the stress effects on live fish more quickly during the recovery period and enabled fish to gradually recover to the state before transportation. Live transportation under low temperatures can inhibit enzyme activity in fish to some extent. Moreover, the application of MO and anesthetics demonstrated an excellent antioxidant enzyme activity effect under low temperatures.

The total antioxidant capacity (T-AOC) refers to the total antioxidant level composed of various antioxidant substances and antioxidant enzymes to protect cells and the body from oxidative stress damage caused by reactive oxygen species. The total antioxidant capacity can be used to evaluate the antioxidant capacity of biologically active substances [49]. Figure 3D indicates that the level of total antioxidant capacity decreased along with the increase in the simulated transport time. The total antioxidant capacity of the control declined dramatically with a significant difference compared with the other treatment groups (*p* ≤ 0.01) during live transport. The total antioxidant capacity of sea bass decreased with the increase in the MO concentration in the transport water during live transport. After 12 h of recovery, the total antioxidant capacity level of samples in the 40 MO group reached the highest level (0.59 U/mL) in comparison with the other groups. Thus, it was found that the addition of MO to the transport water had sedative and antioxidant stress effects during live fish transport and protected fish from oxidative stress. The recommended concentration of MO is 40 mg/L.

### 3.5. Kidney Metabolism Index

**Figure 4:**
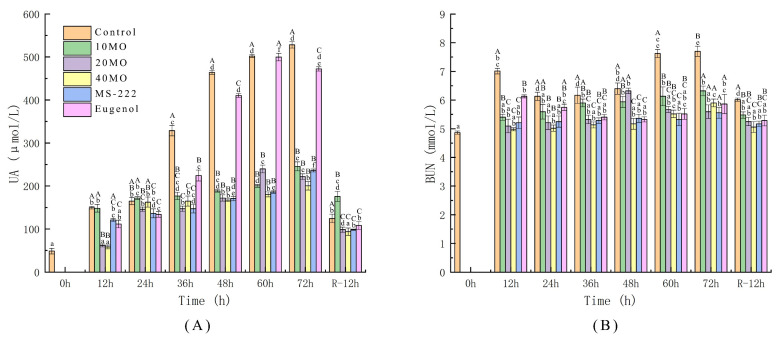
The activity changes in UA (**A**) and BUN (**B**). 10 MO: 10 mg/L MOEO, 20 MO: 20 mg/L MOEO, 40 MO: 40 mg/L MOEO, MS-222: 30 mg/L MS-222, eugenol: 20 mg/L eugenol, control: no agent addition. Vertical bars indicate the standard deviation; Means with different lowercase letters indicate a significant difference between the time intervals within each group (*p* ≤ 0.01) while different capital letters indicate significant differences between groups in each time interval (*p* ≤ 0.01).

Changes in the uric acid level can fully reflect the conditions of metabolism and the immune ability of animals. Serum urea nitrogen is filtered out by the glomerulus and is the main product of nitrogen organic matter and protein metabolism. However, the kidney is susceptible to invasion by various factors and reduction of the waste excretion function, leading to harmful toxins entering the body that cannot be excreted to the outside of the body normally. This leads to an increase in uric acid and urea nitrogen in the blood [50,51]. Figure 4 depicts the changes in uric acid and urea nitrogen in the different treatment groups during live transport of sea bass. It is obvious that the uric acid and urea nitrogen levels of the samples in the control were higher than those of the other groups (*p* ≤ 0.01) while the addition of MO and anesthetics to the transport water reduced this increase for fish to different degrees. The growth rate of uric acid and urea nitrogen slowed down along with the increase in the MO concentration. The uric acid level of samples in the 40 MO group (200.59 μmol/L) and the urea nitrogen level of samples in the MS-222 group (5.90 mmol/L) were at the lowest level, but no differences were observed among the 10 MO, 20 MO, 40 MO, and MS-222 groups after transport for 72 h. The application of MO and MS-222 had similar effects in alleviating kidney metabolism damage during live fish transport. After 12 h of recovery, the uric acid and urea nitrogen levels in fish showed decreasing trends, and the uric acid level of samples in the 40 MO group (93.77 μmol/L) reached the level of samples at 0 h (48.66 μmol/L) with no significant difference. The urea nitrogen level of samples in the 10 MO (5.48 mmol/L), 20 MO (5.23 mmol/L), 40 MO (5.06 mmol/L), MS-222 (5.17 mmol/L), and eugenol (5.29 mmol/L) groups reached the level of samples at 0 h (4.87 mmol/L) with no significant difference. To summarize, as discussed in this section, it was proved that the addition of 40 mg/L MO to the transport water played an important role in the antistress response of live fish.

## 4. Conclusions

The addition of anesthetics and sedatives to transport water is effective and traditional methods keep fish alive during long-distance transport. In this study, sea bass were selected as the research subject, and the effects of live transport on its survivability and oxidative stress injury were investigated. Samples treated with MO and anesthetics were protected from oxidative damage compared to the control. The survival rates of sea bass in the 10 MO, 20 MO, 40 MO, MS-222, and eugenol treatment were higher (80%, 96%, 96%, 100%, 95%, respectively) than the control (50%). The 40 MO sample presented lower levels of serum stress indices, cell apoptosis, and kidney metabolism damage. This study preliminarily explored the effects of MO on reducing stress responses of and physiological changes in live sea bass. The application of MO to the transport water enhanced the antistress ability and survivability during live transport. It is essential to explore the anesthetic and sedative mechanism of essential oil and to enhance animal welfare and minimize the physical stress effects during live transport.

## Figures and Tables

**Table 1:** Determination of the survival rates (%) of sea bass in different treatment groups during long-distance live transport.

Method	Live Transport Time/h						
	12	24	36	48	60	72	R-12
10 MO	100	100	100	100	92	83	80
20 MO	100	100	100	100	96	96	96
40 MO	100	100	100	100	100	96	96
MS-222	100	100	100	100	100	100	100
Eugenol	100	100	100	100	100	100	95
Control	100	100	91	87	79	60	50

Note: R-12 in the table means that fish recovered for 12 h after transport.

## Data Availability

All data, models, and code generated or used during the study appear in the submitted article.
